# Augmented reality navigation method for recontouring surgery of craniofacial fibrous dysplasia

**DOI:** 10.1038/s41598-021-88860-x

**Published:** 2021-05-11

**Authors:** Kai Liu, Yuan Gao, Ahmed Abdelrehem, Lei Zhang, Xi Chen, Le Xie, Xudong Wang

**Affiliations:** 1 Department of Oral and Craniomaxillofacial Surgery, Shanghai 9Th People’s Hospital, Shanghai Jiaotong University College of Medicine, Shanghai, China; 2 Shanghai Key Laboratory of Stomatology, Shanghai, China; 3 Institute of Forming Technology and Equipment, Shanghai JiaoTong University, Shanghai, China; 4 Department of Craniomaxillofacial and Plastic Surgery, Faculty of Dentistry, Alexandria University, Alexandria, Egypt; 5 Institute of Medical Robot, Shanghai JiaoTong University, Shanghai, China; 6 Quanzhou Normal University, Fujian, China

**Keywords:** Diseases, Medical research

## Abstract

The objective of this study is to introduce the application of augmented reality (AR) navigation system developed by the authors in recontouring surgery of craniofacial fibrous dysplasia. Five consecutive patients with craniofacial fibrous dysplasia were enrolled. Through three-dimensional (3D) simulation, a virtual plan was designed to reconstruct the normal anatomical contour of the deformed region. Surgical recontouring was achieved with the assistance of the AR navigation system. The accuracy of the surgical procedure was assessed by superimposing the post-operative 3D craniomaxillofacial model onto the virtual plan. The pre-operative preparation time and operation time were also counted. In all patients, AR navigation was performed successfully, with a mean ± SD of the errors of 1.442 ± 0.234 mm. The operative time of the patients ranged from 60 to 80 min. The pre-operative preparation time was 20 min for each patient. All the patients showed uneventful healing without any complications, in addition to satisfaction with the post-operative aesthetics. Using our AR navigation system in recontouring surgery can provide surgeons with a comprehensive and intuitive view of the recontouring border, as well as the depth, in real time. This method could improve the efficiency and safety of craniofacial fibrous dysplasia recontouring procedures.

## Introduction

Fibrous dysplasia, first described by Lichtenstein in 1938, is defined as an abnormal replacement of normal bone with fibro-osseous connective tissue^1^. Although it is a benign bone lesion, fibrous dysplasia results in destructive functional and aesthetic outcomes when the craniofacial skeleton is involved^2,3^.

Restoration of facial symmetry and normal function is considered a treatment principle for craniofacial fibrous dysplasia^4^. Surgical recontouring, as a minimally invasive procedure, is the preferred treatment^5–7^. However, it is very challenging to successfully achieve the desired results intraoperatively depending on visual exposure and the surgeon's own assessment and experience. At present, although many authors have studied the use of three-dimensional (3D) printing technology, surgical simulation and navigation techniques to assist with the process of surgical recontouring and have achieved some promising clinical effects^8–12^, there are still some difficulties. For example, it is difficult to measure the volume of the lesions to be removed during the recontouring process in real time^4^. In addition, the feedback of important neighbouring structures (nerves, tooth roots, etc.) in the lesion area is usually achieved through repeated intraoperative visual evaluation, while lesions often obscure normal anatomy, making vision calculations unpredictable. Moreover, intraoperative instruments cannot be accurately controlled to operate within the lesions to be removed. Therefore, improper surgical recontouring is likely to lead to unsatisfactory clinical outcomes.

With the continuous development and innovation of digital medicine, augmented reality (AR) has been gradually applied in the medical field and has gained much attention in recent years. Badiali et al.^13^ carried out the Le Fort I osteotomy under the guidance of a video see-through AR system. Gao et al.^14^ completed a study of mandibular angle split osteotomy assisted by an AR system. However, the application of AR navigation to correct craniofacial fibrous dysplasia has not yet been reported in the literature. In our work, we developed an AR navigation technique for recontouring craniofacial fibrous dysplasia to overcome the aforementioned limitations. The technique combines virtually planned and real images into one prospect, enabling the viewing of the imaging, surgical field, and additional information such as the location of the surgical instrument in real time and improving the visualization of every possible narrow space^13,15,16^.

In this clinical series, we describe the application of our AR navigation system in five consecutive patients diagnosed with craniofacial fibrous dysplasia undergoing surgical recontouring. Accordingly, the objective of the current study was to investigate the feasibility of using AR system to enhance the surgical outcome measured by increasing accuracy and decreasing or minimizing errors.

## Materials and methods

Five patients (3 men and 2 women) with unilateral craniofacial fibrous dysplasia were admitted to the Department of Oral and Craniomaxillofacial Surgery at the Ninth People’s Hospital, Shanghai Jiao Tong University School of Medicine, Shanghai, China, from January 2020 to June 2020. The average age was 29 years old (range 18–54 years old). The patients’ characteristics and demographics are thoroughly described in Table 1.

**Table 1:** Patient information.

Patient	Age (y)	Sex	Affected side	Affected area	Follow-up interval (mo)
1	24	F	R	Zygomatic maxillary complex	7
2	54	M	R	Zygomatic maxillary complex	7
3	18	F	R	Zygomatic maxillary complex	3
4	29	M	R	Zygomatic maxillary complex	3
5	20	M	R	Zygomatic maxillary complex	3

*F* female, *M* male, *R* right side, *y* years, *mo* months.

All medical practices followed the Declaration of Helsinki on medical protocols. This study was approved by the Shanghai Ninth People’s Hospital Institutional Review Board (approval number SH9H-2019-T142-1). All methods were performed in accordance with the relevant guidelines and regulations of our hospital. Informed consent was obtained from all the participants. Informed consent has been obtained to publish the information and images in an online open-access publication.

**Figure 1:**
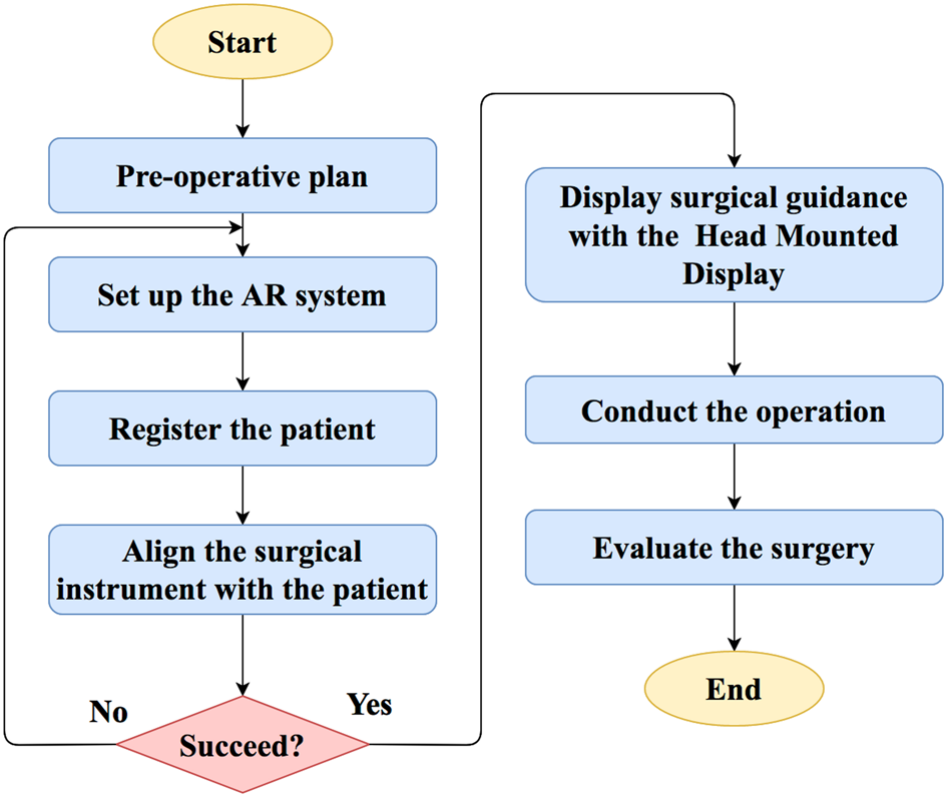
The workflow of the system.

AR-assisted facial recontouring of patients was performed by applying the following workflow, which was implemented by surgeons and biomedical engineers, as shown in Fig. 1.

### STEP 1: preoperative operation plan

A computed tomography (CT) (GE Healthcare, Fairfield, USA) scan was performed pre-operatively, and then the CT images were configured in Digital Imaging and Communications in Medicine (DICOM) format and imported into planning software (ProPlan 2.1, Materialise NV, Leuven, Belgium) to generate a 3D craniomaxillofacial model. For a more accurate placement of the teeth, the dental stone model was scanned with a high-resolution laser surface scanner (Smart Optics AS, Bochum, Germany), yielding a digitalized dental model that can then be merged into the 3D skull model. At this point, we obtained a computerized merged model interpreting highly accurate dental and bony elements^17^. Cranial anatomy was assessed in 3D views and multiple planes (axial, coronal, and sagittal). The normal anatomical contour of the deformed region was mimicked from the normal contralateral side through the mirroring function based on the median sagittal plane as a reference to determine the amount of bone lesion to be removed.

### STEP 2: setup of the AR system and registration

**Figure 2:**
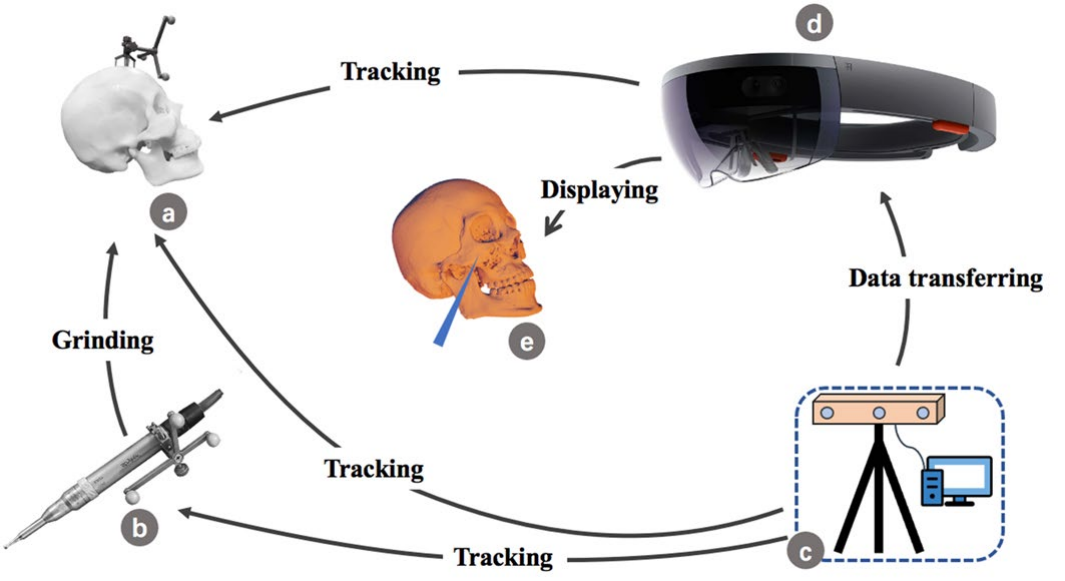
Configuration of the proposed AR navigation system: (**a**) digital reference frame fixed to the patient's craniofacial skeleton; (**b**) surgical drill with clamped digital reference frame; (**c**) optical tracking system and workstation; (**d**) Head Mounted Display; (**e**) 3D virtual planning and the position of surgical drill.

The setup of the AR system consisted of different essential parts for preparation of an integrated image: Head Mounted Display (Hololens, Microsoft Corporation, USA), workstation and optical tracking system (Northern Digital Inc., Ontario, Canada). Figure 2 illustrates the relationship between the different components and system registration, as described below.

A digital reference frame (DRF) firmly fixed to the patient's craniofacial skeleton can be tracked by the optical tracking system and the Head Mounted Display camera, and the surgical drill with another clamped DRF can be tracked by the optical tracking system. The workstation is used to process and update the data of the surgical drill and the patient's craniofacial skeleton’s 3D location and to transfer the data to the Head Mounted Display. The Head Mounted Display mainly played a role in visualizing the hologram, and the 3D virtual planning and the position of surgical drill are displayed through the Head Mounted Display for an intraoperative guidance.

### STEP 3: intraoperative guidance with augmented reality

**Figure 3:**
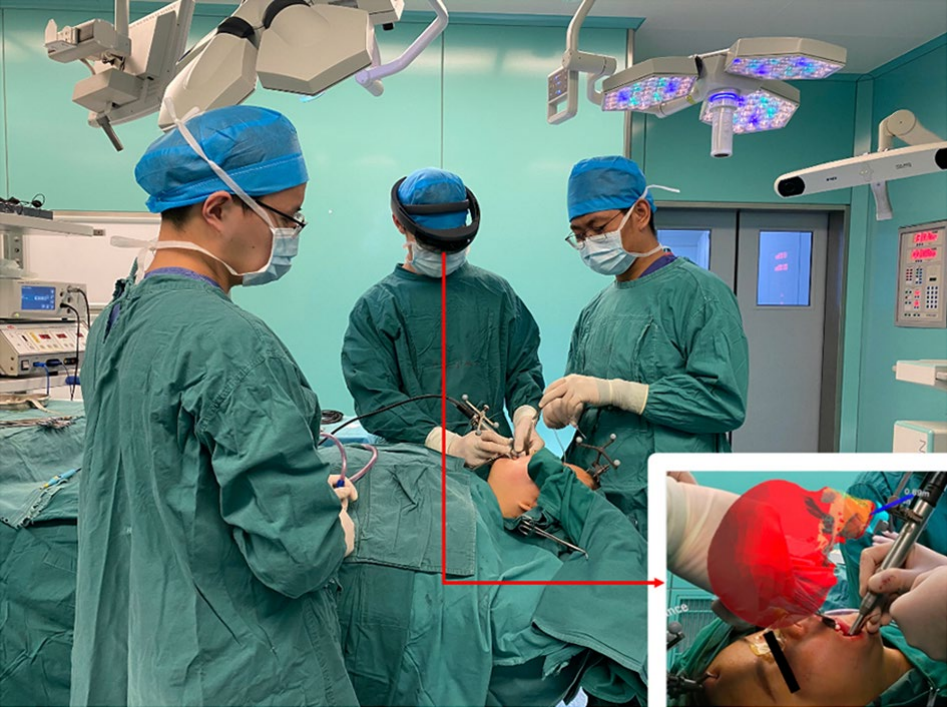
Surgeon carrying out the operation wearing the head mounted display.

**Figure 4:**
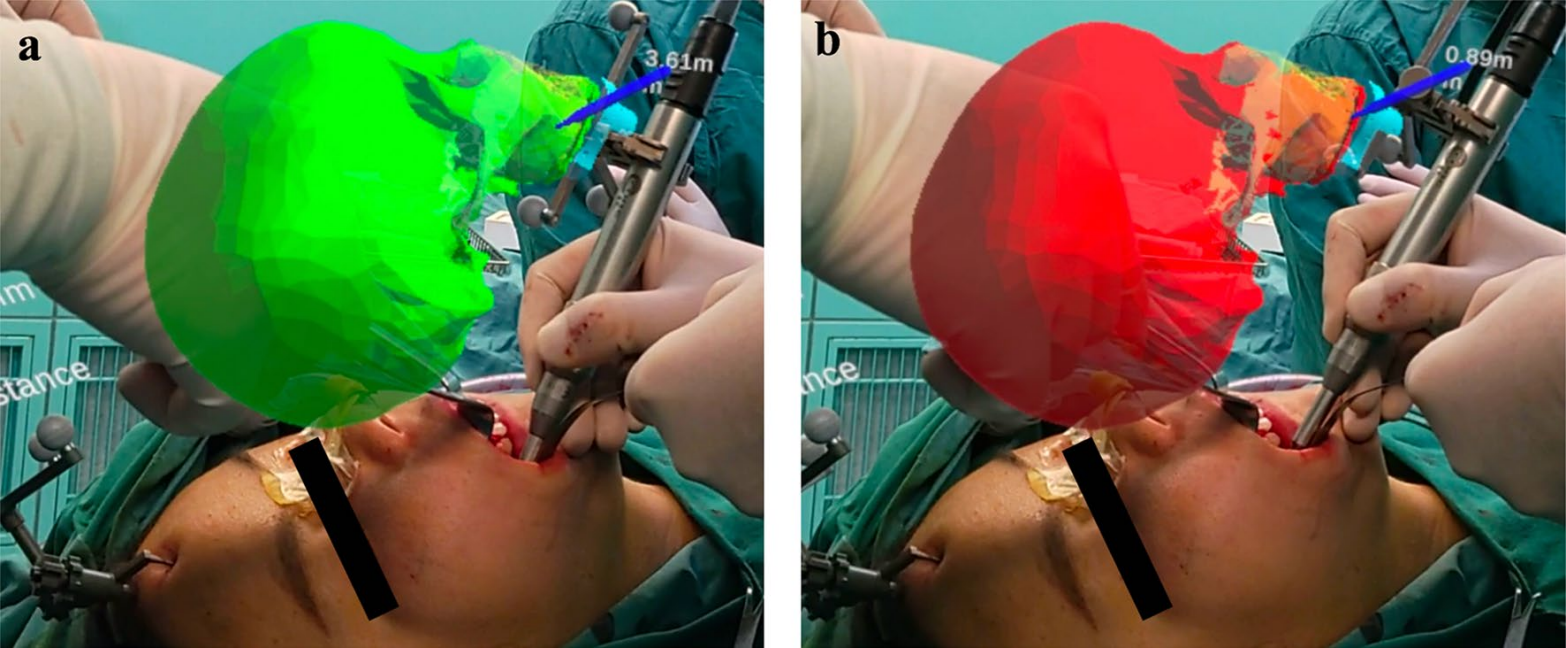
Different stages of the intraoperative guidance: (**a**) When the drill is still at a large distance from the designed surface (> 1 mm), the model is rendered in green, and real-time data will be displayed. (**b**) When the drill is about to reach the designed surface (≤ 1 mm), the model is rendered in red, and real-time data will be displayed.

After the registration step, the surgeon performs the surgery wearing the Head Mounted Display. Following the exposure of the dysplastic zygomaticomaxillary bone, the dysplastic bone was shaved using the surgical drill. With the assistance of the AR navigation system, the surgeon can directly visualize the craniofacial skull and lesion images, surgical field and additional information such as the position of the surgical drill in real time through the head mounted display (Fig. 3). More importantly, the AR system not only ensures that the contouring drill is located in the surgical field for bone contouring but also enables observation of mass resection in real time. Therefore, the surgeon can provide continuous feedback and review the recontouring procedure intraoperatively according to the virtual design. Figure 4 illustrates the precise instrument-based image-guided recontouring procedure guided by real-time visualization through the head mounted display.

The different stages of intraoperative real-time guidance are as follows:

1. When the drill is still at a large distance from the designed surface (> 1 mm), the model is rendered in green, and real-time data will be displayed;
2. When the drill is about to reach the designed surface (≤ 1 mm), the model is rendered in red, and real-time data will be displayed.

### STEP 4: Evaluation of accuracy of the AR system

**Figure 5:**
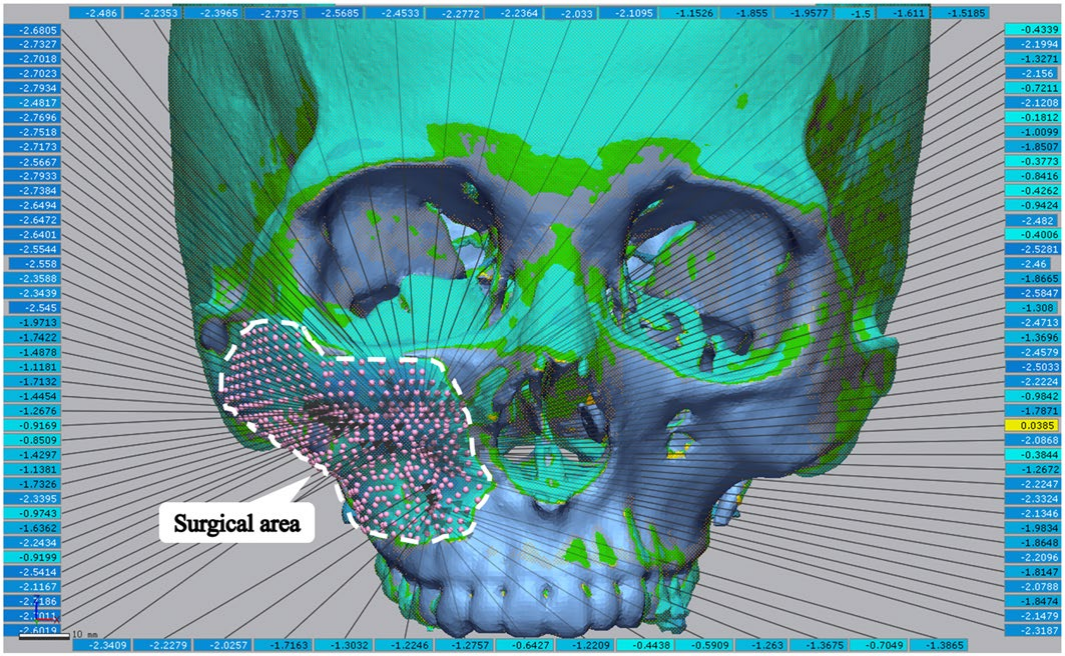
The accuracy evaluation for the performance of the system: The pre-operative virtual plan and post-operative 3D craniomaxillofacial models were imported into 3D analysis software (Geomagic Control X, 3D Systems Inc., South Carolina, USA), and hundreds of pairs of points were generated uniformly in the surgical area. Each of the paired points was located along the normal vector of the skull surface. The distance between the paired points indicated the surgical discrepancy.

CT scanning was performed 3 days following surgery. The accuracy of the surgical procedure was assessed by superimposing the post-operative 3D craniomaxillofacial model onto the pre-operative virtual plan. The detailed evaluation procedure is as follows:

The pre-operative virtual plan and post-operative 3D craniomaxillofacial models are aligned in a common coordinate space. A program was designed using VTK (The Visualization Toolkit, Kitware, Inc., USA) to align the models. By selecting the paired points on the unchanged region of the skull, the planned model’s coordinate space was transformed into that of the post-operative model via point cloud matching. Next, both models were imported into 3D analysis software (Geomagic Control X, 3D Systems Inc., South Carolina, USA) to analyse the superimposed discrepancy. Hundreds of pairs of points were generated uniformly in the surgical area, and the position of each point was obtained, as shown in Fig. 5. Each of the paired points was located along the normal vector of the skull surface. The distance between the paired points indicated the surgical discrepancy.

## Results

Preoperative virtual planning and AR techniques were successfully achieved in all the patients included in the study. Consistency was obtained through superimposition of the post-operative 3D craniomaxillofacial model into the pre-operative virtual plan. The minimum error range of all patients in the anterior of the zygomatic maxillary complex was 0.0053–0.2901 mm. The maximum error range posterior to the zygomatic maxillary complex was 2.4081–3.0903 mm. These findings are consistent with the fact that it is more difficult for the surgeon to recontour posterior lesions of the zygomatic maxillary complex than anterior lesions. The mean ± SD of the error across all patients was 1.442 ± 0.234 mm, indicating that the application of an AR-based navigation technique-assisted craniofacial fibrous dysplasia recontouring system can accurately achieve pre-operative virtual planning and meet clinical requirements (Table 2).

**Table 2:** Errors between the planned and post-operative outcomes (mm).

Patient	Min	Max	Mean ± SD
1	0.0053	2.7934	1.277 ± 0.766
2	0.0881	2.7724	1.218 ± 0.769
3	0.0859	2.9384	1.525 ± 0.992
4	0.2901	3.0903	1.861 ± 0.920
5	0.1113	2.4081	1.326 ± 0.665
Mean			1.442 ± 0.234

**Figure 6:**
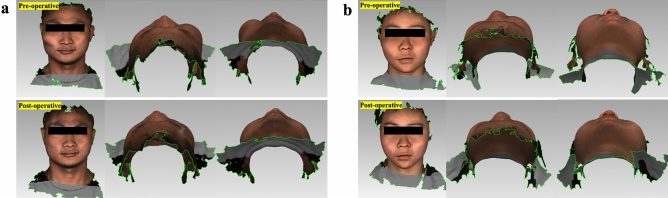
Case presentation: (**a**) A patient (male, 29 years old) with fibrous dysplasia in the right zygomaticomaxillary region. (**b**) A patient (female, 18 years old) with fibrous dysplasia in the right zygomaticomaxillary region.

The operative time of patients ranged from 60 to 80 min. The pre-operative preparation time was 20 min for each patient. The application of the AR navigation technique to assist in craniofacial fibrous dysplasia recontouring reduces the operative time compared with traditional methods. Moreover, all the patients underwent uneventful healing without any complications, and the patients were very satisfied with their post-operative facial appearances (Fig. 6).

## Discussion

For craniofacial fibrous dysplasia, the goal of management is to achieve aesthetic improvement and functional restoration. Radical resection is not feasible for most patients due to the presence of important neurovascular structures located in the craniofacial area and blurring of the lesion boundary. The recontouring procedure is performed by shaving the excess dysplastic bone to achieve facial symmetry and functional restoration. The procedure is minimally invasive and is currently regarded as an optimal substitute in the treatment of craniofacial fibrous dysplasia^2^. However, during the surgical recontouring procedure, it is very challenging to successfully achieve the desired results intraoperatively depending on visual exposure and the surgeon's own assessment and experience. To overcome such challenges, Kang et al.^11^ developed a method of bone recontouring applying 3D printing technology using CT scans and screws as a guide. However, the surgeon might not be able to implant the screws exactly where they should be placed intraoperatively, in addition to the limited number of implants that cannot fully guide bone recontouring. Most importantly, inserting screws in the region near the orbit and neighbouring nerves can be dangerous. Wang et al.^12^ used CT scanning and 3D printing technology to make a template to guide bone recontouring. Although the template can be used to determine the surgical borders, it cannot precisely control the depth of the recontouring procedure.

In recent years, to convey the pre-operative virtual plan to patients with craniofacial fibrous dysplasia, many researchers have used a navigation system^18^. Nevertheless, considering previously studied navigation-guided recontouring techniques, the objective of the navigation process is to corroborate a limited number of points using a probe during the surgical procedure depending on the surgeon’s experience, and by the time too much recontouring is observed, then it is too late to control it. Accordingly, noticeable discrepancies can still be encountered between the pre-operative virtual plan and the real recontouring procedure, and the repeated use of probe verification reduces the surgical efficiency. In addition, the feedback of important neighbouring structures is achieved through repeated intraoperative visual evaluation by the surgeon. However, fibrous dysplasia often obscures normal anatomy, making intraoperative vision assessment unreliable. Currently, AR systems are being developed as a navigation-assisted surgical technique. Due to its enhanced ability to identify physical internal structures and real-time dynamic visualization, AR has gained wide attention^19,20^.

In the present study, we introduce the application of the AR navigation system developed by the authors in craniofacial fibrous dysplasia recontouring surgery. It has the following advantages: first, the surgical drill could be traced by the attaching DRF, thereby resulting in real-time instrument-based visualization while performing the recontouring procedure and setting up a visual cue function according to the distance between the surgical instrument and the designed surface. Second, the 3D image information combined with the pre-operative virtual plan, which is used to identify the border and depth for the recontouring procedure, can not only significantly improve the surgical accuracy but also avoid damaging the important neighbouring structures, such as nerve channels and tooth roots, greatly improving the confidence of operators^21^. Third, the surgical field covered by soft tissue can be viewed intraoperatively from any of the perspective views on the virtual image to reduce the incision range of soft tissue and effectively reduce surgical trauma. Fourth, the AR navigation system reduces the distance between the monitor and the surgeon's eyes compared with traditional navigation, and operators could deeply focus on contouring procedures with better control of surgical procedures.

With the aid of the AR navigation system, operations in the present study were completed smoothly in all cases without complications such as nerve and root injuries. A precise intraoperative transfer of the pre-operative virtual plan to the surgeon’s visualization field is ever more important for craniofacial fibrous dysplasia than for other procedures. In this study, significant matching was obtained by merging the post-operative 3D craniomaxillofacial model onto the pre-operative virtual plan in 5 consecutive patients. The mean error was 1.442 ± 0.234 mm (range 1.218 ± 0.769–1.861 ± 0.920 mm), with little variability. The preliminary results indicated that AR-assisted craniofacial fibrous dysplasia recontouring can accurately achieve pre-operative virtual planning and is feasible for clinical application. The minimum error range of all patients in the anterior of the zygomatic maxillary complex was 0.0053–0.2901 mm. The maximum error range posterior to the zygomatic maxillary complex was 2.4081–3.0903 mm. These findings can be explained by the fact that the surgical instrument located posterior to the zygomatic maxillary complex interferes with the corner of the mouth during the recontouring procedure, which makes it more difficult for the surgeon to operate posteriorly than anteriorly. In addition to the errors caused by the operation itself, the registration accuracy and tracking accuracy may be other important factors affecting the system accuracy. Therefore, the DRF fixed to the patient's forehead must be firm and reliable.

This study introduces the application of an AR navigation system in recontouring craniofacial fibrous dysplasia surgery, which can enable surgeons to better control the operation process, effectively improve surgical efficiency and safety, reduce patient trauma and shorten the operative time. However, the current study still has some limitations. First, the current report was designed to introduce a new technique with the AR system developed by the authors; as such, the sample size and follow-up period should be increased to better analyse the outcomes, which is the issue of upcoming future research in our group. Second, prospective longitudinal clinical studies are needed to obtain more valid results. Third, considering the impact of AR application on surgeons, overly use of AR may cause visual shortness, dizziness, and mental fatigues.

## Conclusion

Using our AR navigation system in the contouring procedure for craniofacial fibrous dysplasia can provide surgeons with a comprehensive and intuitive view of the recontouring border and its neighbouring anatomical structures, as well as the depth for the recontouring procedure in real time. This method could improve the efficiency and safety of the procedure and shorten the operative time. In summary, we believe that AR navigation is an important potential technology for craniofacial fibrous dysplasia recontouring.
